# A meta-analysis of the association between gestational diabetes mellitus and chronic hepatitis B infection during pregnancy

**DOI:** 10.1186/1756-0500-7-139

**Published:** 2014-03-11

**Authors:** Dechuan Kong, Haiyan Liu, Shan Wei, Yan Wang, Anqun Hu, Wenhui Han, Naiqing Zhao, Yihan Lu, Yingjie Zheng

**Affiliations:** 1 Department of Epidemiology, School of Public Health, Fudan University, Shanghai 200032, China; 2 The Key Laboratory on Public Health Safety, Ministry of Education, Fudan University, Shanghai 200032, China; 3 Department of Clinical Laboratory, Anqing City Hospital, Anqing 246003, Anhui Province, China; 4 Department of Gynaecology and Obstetrics, Anqing City Hospital, Anqing 246003, Anhui Province, China; 5 Department of Biostatistics, School of Public Health, Fudan University, Shanghai 200032, China; 6 Department of Sanitary Microbiology, School of Public Health, Fudan University, Shanghai 200032, China

**Keywords:** Chronic hepatitis B infection, Gestational diabetes mellitus, Pregnant women, Meta-analysis

## Abstract

**Background:**

Chronic hepatitis B (CHB) infection during pregnancy is associated with insulin resistance. A meta-analytic technique was used to quantify the evidence of an association between CHB infection and the risk of gestational diabetes (GDM) among pregnant women.

**Methods:**

We searched PubMed for studies up to September 5th 2013. Additional studies were obtained from other sources. We selected studies using a cohort-study design and reported a quantitative association between CHB infection during pregnancy and risk of GDM. A total of 280 articles were identified, of which fourteen publications involving 439,514 subjects met the inclusion criteria. A sequential algorithm was used to reduce between-study heterogeneity, and further meta-analysis was conducted using a random-effects model.

**Results:**

Ten out of the fourteen studies were highly homogeneous, indicating an association of 1.11 [the adjusted odds ratio, 95% confidence interval 0.96 - 1.28] between CHB infection during pregnancy and the risk of developing GDM. The heterogeneity of the additional four studies may be due to selection bias or possible aetiological differences for special subsets of pregnant women.

**Conclusions:**

These results indicate that CHB infection during pregnancy is not associated with an increased risk of developing GDM among pregnant women except those from Iran.

## Background

The hepatitis B virus (HBV) accounts for significant morbidity and mortality worldwide [1]. An estimated one-third of the worldwide population has been exposed to HBV, and 400 million people are chronic carriers [1]. HBV can be transmitted vertically, sexually, and through other routes, and pregnant women play important roles in each of those transmission routes. Chronic hepatitis B (CHB) infection during pregnancy often results in hepatic flare [2] and possibly other adverse outcomes [3-17], including gestational diabetes mellitus (GDM)[3-8,10-13,15-17], although conflicting evidence exists on this last point.

GDM is defined as glucose intolerance with an onset or first recognition during pregnancy. Asian women, including Chinese women, have been found to have the highest incidence of GDM [18]. Women with GDM are at an increased risk for adverse perinatal and maternal outcomes including macrosomia, caesarean section, birth trauma, and diabetes later in life, although treatment significantly decreases these risks [19]. Until now, liver disease of various aetiologies has been implicated as a cause of diabetes [20], and chronic hepatitis C virus (CHC) infection has been established to increase the risk of diabetes mellitus (DM) [21].

Similarly to CHC infection, CHB infection is considered to be associated with insulin resistance (IR) [22], which implies that it may increase the risk of developing DM and/or GDM. However, the association between CHB infection and the risk of DM and/or GDM remains less convincing. An increased risk was reported for DM only by Li-Ng [23], but this result was not confirmed by a recently published ten-year cohort study [24]. Several studied have reported an increased risk for GDM [4,6,7,10,11], but this result was contradicted by many others [3,5,8,9,12-17]. Due to the limited resources and time available for HBV vaccination, the prevalence of CHB infection among pregnant women remains high, especially in HBV epidemic areas such as China [25].

To address these issues, we performed a meta-analysis of cohort studies to investigate whether CHB infection during pregnancy is associated with an increased risk of GDM.

## Methods

### Data sources and search strategy

This meta-analysis was conducted in compliance with the Meta-Analysis of Observational Studies in Epidemiology guidelines (http://edmgr.ovid.com/ong/accounts/moose.pdf). Two independent investigators (Kong and Liu) searched PubMed from 1966 to September 5th, 2013 using the combinations of terms “hepatitis B” or “hepatitis B surface antigen (HBsAg)” and “pregnancy outcome” or “prenatal outcome” or “perinatal outcome” or “gestational diabetes”. We sifted through potentially relevant articles, firstly by titles and abstracts, and then we retrieved the full texts of articles for detailed review. Further, we scanned the reference lists of the articles that met the inclusion criteria in our analysis, and searched for those articles or citations in the Web of Knowledge, Google Scholar and Google to obtain additional studies.

### Inclusion and exclusion criteria

Articles were included if they used a cohort design and reported a quantitative association between CHB infection during pregnancy and the risk of GDM among pregnant women versus a non-CHB control group. For studies that enrolled overlapping pregnant women, only more recently published studies and/or those with larger sample sizes were included.

Studies included in this analysis defined CHB infection status during pregnancy by the presence or absence of hepatitis B surface antigen (HBsAg) in blood during the first prenatal care visit, on admission to the labour ward, or before delivery. GDM diagnosis was performed according to the recommended clinical practice.

### Data extraction

A form designed a priori was used to extract the information from the included studies. Two independent investigators (Han and Wei) performed the data extraction. A third investigator (Wang) examined the results, and a consensus was considered as agreement between at least two out of the three investigators. GDM was the primary outcome measure. We extracted the following data (Table 1): authors’ name, journal and year of publication, country of origin, enrolment period, incidence of GDM in pregnant women who had CHB infection and those who did not, general characteristics of pregnant women, unadjusted and/or adjusted effect size and the adjusted variables if available.

**Table 1:** General characteristics and quality scores of the 14 studies included in this meta-analysis

**No**	**Author, publication year, design (matched variables), study site, country, PW (no, HBsAg +%) , enrolled period (and NOS)**	**Inclusion/exclusion**	**Identification of HBsAg/GDM (data retrieval)**	**Baseline comparison between HBsAg + and HBsAg- PW**		**Effect estimation (95% CI)/adjustment variables**
				**Similarity**	**Dissimilarity**	
1.	Wong SF, 1999	PW delivered after 24 GW or babies with BW ≥ 500 g/AHP, non-SP, acute pelvic inflammatory disease, STI, smoker or illicit drug users, and DM	ELISA/Australian criteria (computer database)	Mean age, parity, history of abortion	NA	OR_c_ = 1.05 (0.69, 1.61)
	RCS, with all HBsAg- as controls					
						OR_a_ = 1.00 (0.56,1.76)^*1^/NA
	Princess Margaret Hospital					
	Hongkong, China					
	SP (7105, 11.6%)					
	Jul.1,1996-Aug.31,1998 (6)					
2.	Lao TT, 2003	Chinese PW, recruited at 28-30 GW, haemoglobin concentration ≥10 g/dl and mean cell volume ≥ 80 fL at the initial visit at or before 14 GW/antenatal visit after 14 GW, anemia or hemoglobinopathy, GDM diagnosed before 28 GW, non-SP	ELISA/WHO criteria (medical record)	Weight, height, BMI, parity, socioeconomic status	The HBsAg + PW were significantly older, with more OGTTs performed, with higher iron status, with higher prevalence of past obstetric history, family or medical history and had an advanced age (≥35 years)	RR_c_ = 2.97 (2.00, 4.42)
	RCS, with a sample as controls					OR_c_ = 3.94 (2.27, 6.84)
	Queen Mary Hospital or others					RR_a_ = 3.51 (1.83, 6.73)/Age, BMI, socioeconomic status, parity and presence of significant obstetrics, family and past history.
	Hongkong, China					
	SP (767, NA)					
	Over four months (5)					
3.	To WWK, 2003	PW delivered after 24 GW/AHP(2); incomplete or contradicting data(152)	ELISA/Australian criteria (computer database)	Age, GW at delivery, BMI, parity, incidence of non-SP or STIs; no clinical manifestations of liver disorders or hepatitis.	A higher prop. of HBsAg + PW from mainland China	OR_c_ = 0.81 (0.59, 1.12 )
	RCS, with all HBsAg- as controls					
						OR_a_ = 0.77 (0.47, 1.26)^*1^/NA
	United Christian Hospital					
	Hongkong, China					
	AP (13946, 9.72%)					
	Jan. 1997-Dec. 2000 (6)					
4.	Tse KY, 2005	NA/incomplete data	ELISA/WHO criteria (medical record)	BMI, height, weight and Hb level at booking, ethnic, history of stillbirth, IVF pregnancy, haemoglobin level at booking, past medical history	Among the HBsAg + PW, the weight gain in those with GDM was significant less than those without GDM.	OR_c_ = 1.89 (1.14, 3.13)
	RCS, matched for age and parity					
						OR_a_ = 2.04 (1.21, 3.44)/Age, weight and parity
	Queen Mary Hospital (section mainly for high-risk paturients)					
	Hongkong, China					
	SP (3348, 7.56%)				Among the HBsAg- PW, the weight gain in those with GDM was non-significant less than those without GDM.	
	Jan. 2000-2002 (7)					
5.	Lao TT, 2007	SP, 99% of those PW delivered at or after 24 GW/incomplete data (147)	ELISA/ WHO criteria^*4^ (computer database, ICD coding)	Age, height, parity and history of DM	The HBsAg + PW had lower weight and BMI, with lower prop. of overweight and smokers, and with higher prop. of Asian origin, married, unemployed and history of induced abortion.	OR_c_ = 1.31(1.08, 1.57)
	RCS, with all HBsAg- as controls					
						OR_a_ = 1.24 (1.01, 1.51)/parity, age, BMI, presence of iron deficiency anaemia, and smoking.
	Queen Mary Hospital					
	Hongkong, China					
	SP (14464, 8.3%)					
	1998-2001 (8)					
6.	Lert-amornpong S,2007	Healthy PW Age 20-39 years old/chronic illness, non-SP, HIV+, smoker, alcohol drinker	ELISA/NDDG (medical record)	Age, weight at booking, weight gain, hematocrit at booking, history of contraception, parity and past health.	NA	OR_c_ = 3.04 (0.60, 15.28)
	RCS, matched for age and date of delivery					
						OR_a_ = 2.89 (0.55, 15.17)^*1^/NA
	Phramongkutklao College of Medicine					
	Bangkok,Thailand					
	SP (8515, 1.93%)					
	Jan.1, 2003-Dec.31,2005 (5)					
7.	Thungsuk R, 2008	Healthy PW	ELISA/ADA (medical record)	Age, hematocrit at booking, parity and past health	NA	OR_c_ = 1.39 (0.37, 5.28)
	RCS, matched for age, parity and year of delivery	Age 20-39 years old/chronic illness^,^ non-SP, HIV+, smoker, alcohol drinker				
						OR_a_ = 1.32 (0.33, 5.29)^*1^/NA
	Sawanpracharak Hospital					
	Nakhonsawan, Thailand					
	AP (2548, 1.3%)					
	Jan. 2005-Dec, 2007 (5)					
8.	Saleh-Gargari S, 2009	SP/AHP	ELISA/ADA (medical record)	Age, parity, BMI, hemoglobin level at admission, past health history	NA	OR_c_ = 4.13 (1.96, 8.70)
	RCS, matched for age, parity, and BMI					
						OR_a_ = 3.62 (1.60, 7.90)/NA
	the labor ward in Mahdieh and Vali Asr Tertiary Care Hospital					
	Tehran, Iran					
	SP (NA, NA)					
	Mar. 2001-Dec. 2008 (6)					
9.	Aghamohammadi A, 2011	SP/	ELISA/ADA (medical record)	Age, parity, haemoglobin level at booking, past medical history	NA	OR_c_ = 2.34 (1.32, 4.17)
	RCS, matched for age and parity, selected at random	AHP				OR_a_ = 1.53 (1.19, 1.97)/NA
	the labor ward in Imam Khomeyni					
	Sari, Iran					
	SP (2953, 5.07%)					
	Jan.,2005-Dec.,2008 (6)					
10.	Lobstein S, 2011	SP (8193)/non-SP (427); HBsAg not available (887)	ELISA/IADPSG (computer database)	BMI, age, history of stillbirths, ectopic pregnancies, IVF pregnancies	The HBsAg + PW had lower weight and height, higher prop. of Asian origin, married, unemployed, history of induced abortion, and lower prop. of primipara	OR_c_ = 2.82 (0.17, 46.68)
	RCS, with all HBsAg- as controls					
						OR_a_ = 2.20 (0.13, 37.03)^*1^/NA
	Gynecological University Hospital					
	Leipizig, Germany					
	AP (9507, 0.48%)					
	Jan 1, 2001-Dec. 31,2006 (6)					
11.	Reddick KL, 2011	NA/DM, HCV	ELISA/ADA	NA	The HBsAg + PW were younger, with more black and Asian, and higher prop. of public-assisted insurance, any substance use, any STI and medical complications	OR_c_ = 1.78 (1.27, 2.50)
	RCS, with all HBsAg- as controls					
						OR_a_ = 1.39 (0.88, 2.12)/age, race, insurance status, substance use, STI and medical complications
	1054 hospitals					
	37 states, USA					
	AP (297664, 0.27%)					
	1995–2005 (8)					
12.	Lu YP, 2012	SP/pre-existing diabetes, impaired glucose tolerance, hypertension, etc; syphilis, HIV; co-infected or super-infected with HCV, HDV, HAV, HEV and other infections, smokers drug users, alcohol drinkers and prescription users.	ELISA/ADA (medical record)	Age, height, BMI before pregnancy, parity, times of pregnancy, GW	NA	OR_c_ = 0.90 (0.48, 1.67)
	RCS, with a random sample of all HBsAg- as controls					
						OR_a_ = 0.85 (0.41, 1.77)^*1^/NA
	First Affiliated Hospital of Jinan University					
	Guangzhou,,China					
	SP (NA, NA)					
	May 2009-Jul. 2011 (6)					
13.	Lao TT, 2013	PW delivered at or after 24 GW/NA	ELISA/Australian criterion (computer database, ICD coding)	Height and medical history	The HBsAg + PM were insignificantly younger, and with higher prop. of overweight, BMI and multiple parity	OR_c_ = 0.96 (0.88, 1.04)
	RCS, with all HBsAg- as controls					OR_a_ = 0.91 (0.62, 1.34)^*1^/NA
	Princess of Wales Hospital					
	Hongkong, China					
	SP (86537, 10.0%)					
	Jan. 1995-Dec. 2009 (5)					
14.	Mak SL, 2013	PW delivered after 24 GW	ELISA/Australian criterion (computer database)	Maternal condition and past medical health	Parity was higher in HBsAg + PW	OR_c_ = 0.97 (0.74, 1.28)
	RCS, with all HBsAg- as controls					
						OR_a_ = 0.92 (0.58, 1.48)^*1^/NA
	Queen Elizabeth Hospital					
	Hongkong, China					
	AP (9526, 7.85%)					
	Oct. 1^st^, 2010- Dec. 31^st^, 2011 (6)					

Notes.

PW: pregnant women; NOS: Newcastle-Ottawa Quality Assessment Scale; no: number; HBsAg: hepatitis B virus surface antigen; GDM: gestational diabetes mellitus; CI: confidence interval; RCS: retrospective cohort study; SP: single pregnancy; GW: gestational weeks; BW: birth weight; AHP: acute hepatitis during pregnancy; STI: sexually transmitted infection; DM: diabetes mellitus; ELISA: Enzyme-linked immunosorbent assay; NA: not available; OR_c_: crude odds ratio; OR_a_: adjusted odds ratio; WHO: World Health Organization; BMI: body mass index; OGTT: oral glucose tolerance test; RR_c_: crude risk ratio; RR_a_: adjusted risk ratio; Hb: haemoglobin; IVF: In vitro fertilization; prop: proportion; HIV: human immunodeficiency virus; NDDG: National Diabetes Data Group; ADA: American Diabetes Association; IADPSG: International Association of Diabetes in Pregnancy Study Group; HCV: hepatitis C virus; HDV: hepatitis D virus; HAV: hepatitis A virus; HEV: hepatitis E virus; ICD: International Classification of Diseases.

^*1^OR_a_ was calculated using external estimates of confounding by the method of coefficient adjustment.

### Assessment of methodological quality

Two independent investigators (Kong and Hu) evaluated the quality of each study using the Newcastle-Ottawa Quality Assessment Scale (NOS) [26]. The third investigator (Lu) examined the results, and a consensus was considered as agreement between at least two out of the three investigators (Table 1). We did not consider the 2nd item of the NOS scale (Was follow-up long enough for outcomes to occur) or the 3rd item (Adequacy of follow up of cohort) because the final outcomes of all pregnant women were followed up to their delivery dates. We defined studies with NOS ≥ 7 as high quality and those with NOS < 7 as low quality.

### Statistical analysis

#### *Effect measurement: odds ratio and its adjustment*

The cross-study effect of CHB infection vs. non-CHB control on the risk of GDM was measured using the odds ratios (OR) with 95% confidence intervals (CIs), which were reported as the effect measurements in all of the included studies except for one [4], which reported relative risk.

The adjusted OR (OR_a_) was automatically included in our analysis; the unadjusted or crude OR (OR_c_) was corrected using external estimates of confounding by the method of coefficient adjustment introduced by Greenland [27]. Briefly, the confounding effect U = OR_c_/OR_a_ was calculated by an external study. The variance of U was estimated using the formula V_u_ = V_a_-V_c_, where V_a_ and V_c_ are the variances of the natural logarithm of the OR_c_ and OR_a_ from the same study, respectively. Then, we used U and its variance V_u_ for further correction. In our analysis, the OR_c_ from the Lobstein study [12] and the Chinese or Thailand studies [3,5,8,9,15-17] were adjusted using the estimates of U and V_u_ from Reddick 2011 [13] and Lao 2007 [7], respectively.

#### *Heterogeneity analysis and its sensitivity analysis*

We used a random-effects model to pool the OR_a_ across studies in Stata version 10.0 (Stata Corp). Heterogeneity was first explored by a *χ*^2^ test with a *P* value <0.10 considered statistically significant and then judged visually by a forest plot, funnel plot and Galbraith plot. The extent of heterogeneity was measured by Higgins’ I^2^. We also explored the influence of heterogeneity on the pooled OR_a_ across studies using a sensitivity analysis introduced by Patsopoulos [28]. Briefly, for a meta-analysis of n studies, we perform n new meta-analyses where one study is excluded from the calculation each time. The study that leads to the largest decrease in I^2^ upon exclusion is dropped, and a new set of n-1 studies is created. This process is continued until the I^2^ is decreased to the lowest possible value.

For the heterogeneous studies that were excluded from further analysis, we explored the possible reasons for the heterogeneity using a causal diagram.

#### *The combined effect estimate*

For the homogenous studies, the Dersimonian and Laird random-effects model was used to pool the OR_a_ across studies in Stata version 10.0 (Stata Corp). Sub-group analysis and meta-regression were carried out to examine the effect in relation to design, type of epidemic area, article quality and GDM diagnostic criteria. We used the interaction test to estimate the difference between two sub-groups [29]. Publication bias was assessed by a visual inspection of a funnel plot and by the Egger’s and Begger’s test [30], and a nonparametric trim and fill method was applied if possible [31]. The predictive intervals were calculated using a t-distribution with k-2 degrees of freedom, where k represents the number of studies [32].

## Results

### Literature search

The literature search retrieved 280 papers, 29 of which were identified as potentially relevant to the current analysis. Further backwards or citation searches produced six additional papers. Only fourteen papers [3-13,15-17] representing fourteen independent studies were included in our analysis. The reasons for exclusion of the other studies are listed in Additional file 1: Figure S1.

### General characteristics about the studies included in meta analysis

The fourteen studies included in the present analysis were published between 1999 and 2013 (Table 1). Two studies each were conducted in Thailand [8,9] and Iran [10,11], one in Germany [12], the United States [13], and mainland China [15], and the other seven in Hong Kong, China [3-7,16,17]. All fourteen were hospital-based retrospective cohort studies.

The CHB infection status was determined by the presence or absence of HBsAg during pregnancy, and the diagnosis of GDM was determined through routine clinical practices. This information was retrieved directly from medical records or indirectly from medical records based on a computerised database through the International Classification of Diseases codes.

### Heterogeneity analysis and its sensitivity analysis

The high heterogeneity of the OR_a_ across the fourteen studies was demonstrated by visual observation of the forest plot, funnel plot, and Galbraith plot and further verified by the Higgins’ I^2^ value of 58.9% (*χ*^2^ = 31.6, df = 13, *p = 0.003*).

**Figure 1:**
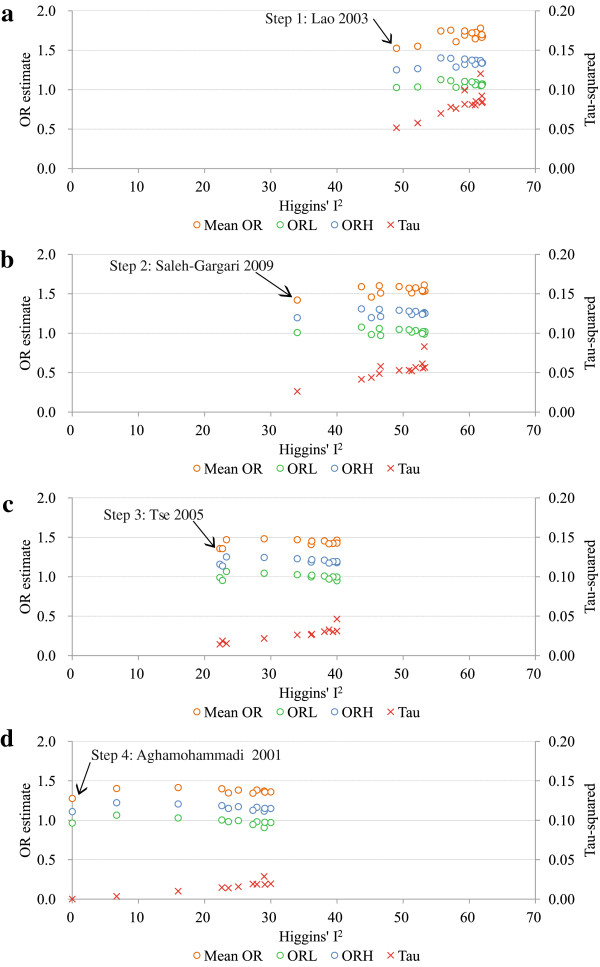
**Pooled odds ratios, their 95% confidence intervals, Tau-squared and Higgins’ I**^**2 **^**values for the association between chronic hepatitis B infection during pregnancy and the occurrence of gestational diabetes mellitus using a random-effects model based sequential algorithm.** OR: odds ratio; ORL: 95% lower confidence limit of OR; ORH: 95% higher confidence limit of OR; Notes: In each step of the analysis, one study was removed out of: **(a)** the original fourteen studies (Step 1); **(b)** the remaining thirteen studies after the exclusion of the Lao 2003 (Step 2); **(c)** the remaining twelve studies after the exclusion of Lao 2003 and Saleh-Gargari 2009 (Step 3); **(d)** the remaining eleven studies after the exclusion of Lao 2003, Saleh-Gargari 2009 and Tse 2003 (Step 4). The arrows point to the studies whose removal minimises the Higgins’ I^2^ value. The blue, green and orange hollow circles denote the pooled OR_a_, 95% ORL and ORH, respectively, and the red multiple signs denote the Tau-squared values.

I^2^ was reduced by removing one study at a time using a random-effects model based sequential algorithm [28]. As seen from Figure 1a-d, removing one study each time led to a gradual decrease in the Tau-squared value with decreasing I^2^, and the 95% CIs of the pooled OR_a_ across studies became narrower, with the means, lower and upper 95% CIs ranging from 1.00 - 1.40, 0.82 - 1.13, and 1.21 - 1.78, respectively. Actually, 38% of the 95% lower CIs were below 1.0 during this process.

**Figure 2:**
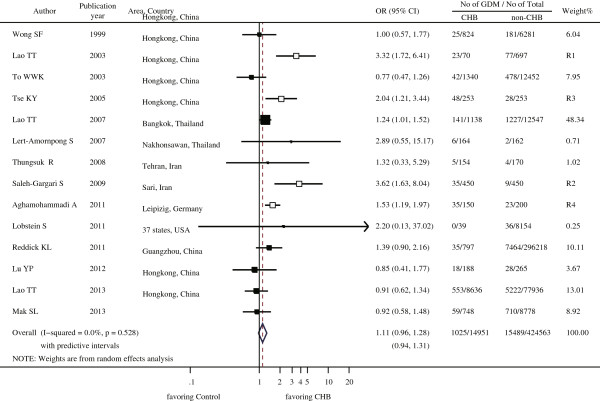
**Effect of chronic hepatitis B infection during pregnancy on the occurrence of gestational diabetes mellitus using a random-effects model.** OR: odds ratio; CI: confidence interval; CHB: chronic hepatitis B infection; GDM: gestational diabetes mellitus. Notes: The black boxes denote the ten studies used for this analysis; the hollow boxes with the same size denote the four studies that were excluded from the analysis. R1, R2, R3 and R4 denote the studies in the order of their removal during the process for the reduction of Higgins’ I^2^.

After four studies were removed (Lao 2003 [4], Saleh-Gargari 2009 [10], Tse 2003 [6] and Aghamohammadi 2011 [11]), the remaining ten studies were highly homogeneous, with a Higgins’ I^2^ value of 0.0% (*χ*^2^ = 8.06, df = 9, *p* = 0.53, Figure 2) and a Tau-squared value of 0.0.

### The combined effect estimate

The ten highly homogeneous studies involved 99.4% (436,991/439,514) of the subjects among the fourteen studies, and together reported a GDM prevalence of 6.30% (884/14,028) among pregnant women with CHB infections and 3.63% (15,352/422,963) among uninfected pregnant women. The association between CHB infection during pregnancy and the risk of developing GDM was 1.11 [the adjusted odds ratio (OR_a_), 95% CIs: 0.96 - 1.28, 95% predictive intervals 0.94 - 1.31] (Figure 2). These findings suggest that CHB infection during pregnancy does not increase the risk of GDM, in agreement with all of the individual studies except for one [7], which suggests that CHB infection during pregnancy is associated with a higher risk of GDM.

Subgroup and meta-regression analysis showed that the pooled OR_a_ across studies could not be modified by the following characteristics: quality of papers (high vs. low), designs (all non-CHB pregnant women as controls vs. an unmatched or matched sample of non-CHB pregnant women as controls), epidemic levels (high, middle vs. low), and GDM diagnostic criteria.

**Figure 3:**
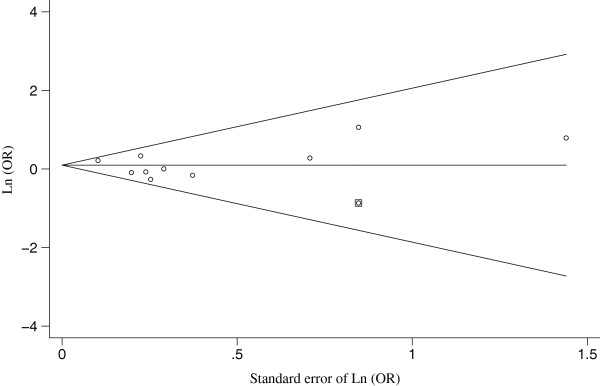
**The Egger’s funnel plot with pseudo 95**% **confidence limits for the ten homogenous studies analysing the effect of chronic hepatitis B infection during pregnancy on the occurrence of gestational diabetes mellitus by using trim and filled methods.** OR: odds ratio; Ln: natural logarithm. The hollow circles with or without enclosed boxes represented the original and filled studies, respectively.

Visual inspection of the funnel plots of the OR_a_ from the ten studies demonstrated a possible publication bias. After trim and fill methods were performed, the pooled OR_a_ was 1.10 (95% CIs: 0.96 - 1.26; 95% predictive intervals: 0.94 - 1.30) (Figure 3).

### Possible reasons for the heterogeneity of the remaining four studies

**Figure 4:**
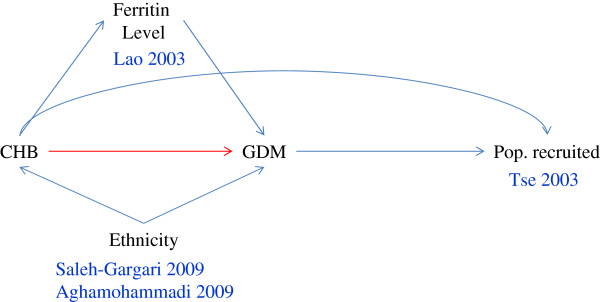
**Possible causal diagram related to the four excluded studies analysing the relationship between chronic hepatitis B infection during pregnancy and gestational diabetes mellitus.** CHB: chronic hepatitis B infection; GDM: gestational diabetes mellitus; Pop: population. The arrow represents a causal relationship between the two variables at both edges of the arrow. The variable that the arrow points to denotes the outcome of the variable at the other end of the arrow. The red arrow denotes the relationship under study. The blue arrow lines denote the other variables that could affect this relationship. The author names and publication years are associated with the variables as appropriate.

The four heterogeneous studies that were excluded from further analysis all reported a higher risk of GDM for pregnant women with CHB infection (Figure 2). The effect of their removal on the heterogeneity was depicted within a causal diagram (Figure 4). The first and third excluded studies, Lao 2003 [4] and Tse 2003 [6], respectively, had both recruited a special subset of pregnant women from the population in Hong Kong, China. For the former study, CHB infected pregnant women were subjected to more rigorous oral glucose tolerance testing (OGTT), and exhibited a higher serum ferritin concentration which was considered to be both an outcome of the CHB infection and a risk factor of GDM [33]. The pregnant women in the latter study had multiple risk factors, with some suffering from significant medical diseases requiring active treatment, including pre-existing diabetes mellitus. Both CHB infection during pregnancy and GDM may lead to a high-risk status among pregnant women, which would affect their recruitment into the study. Thus, both studies may have enrolled more CHB infection-related GDM [34], a selection bias resulting in the overestimation of the association between CHB infection and GDM. The second and fourth excluded studies, Saleh-Gargari 2009 [10] and Aghamohammadi 2009 [11], respectively, were both conducted in Iran with similar designs. As we know, the prevalence of both CHB infection and GDM differs among ethnic groups [18], making it possible that the CHB infection-GDM association may exist in pregnant Iranian women but not the others. These issues should be addressed further in future studies.

## Discussion

Our results suggest that CHB infection during pregnancy does not represent a significant general risk for the development of GDM as compared to non-CHB infected controls.

The high initial between-study heterogeneity in this meta-analysis prevented us from drawing strong conclusions. To overcome this problem, we firstly used a sequential algorithm based sensitivity analysis of between-study heterogeneity [28], which differentiated those homogenous studies from the other heterogeneous ones. This strategy was helpful in identifying and sequentially excluding the heterogeneous studies, i.e., at least those with outliers, which made the analysis based on the random-effects model more reasonable [32]. Although the acceptable level of heterogeneity remains unknown [35], we were conservative and chose the lowest Higgins’ I^2^ as our standard for this differentiation. As a result, this strategy resulted in a parallel decrease in both Higgins’ I^2^ and Tau-squared, with values approaching 0.0% and 0.0, respectively. Finally, we differentiated ten homogenous studies from the remaining four heterogeneous ones.

Those ten homogeneous studies reported GDM prevalences of 6.30% and 3.63% among pregnant women with and without CHB infection, respectively. The risk of GDM for CHB-infected pregnant women was comparable to the risk for uninfected women (OR_a_ = 1.11, 95% CI: 0.96-1.28). These findings suggest that CHB infection during pregnancy is not associated with an additional risk of GDM.

Two of the heterogeneous studies, Lao 2003 [4] and Tse 2003 [6], provided further support. The study of Lao 2003 [4] enrolled pregnant women with higher levels of ferritin, which independently predicts the severity or advanced fibrosis of non-alcoholic fatty liver disease [36]. The study of Tse 2003 [6] enrolled high-risk pregnant women. Thus, both of these studies may have enrolled women with more progressive stages of CHB infection, which would have resulted in the overestimation of the association between CHB infection and GDM.

It remains unclear why only Iranian pregnant women with CHB infection appear to have an increased risk of GDM. Both Iranian studies, Saleh-Gargari 2009 [10] and Aghamohammadi 2011 [11], were conducted on Iranian pregnant women with similar designs [10,11] but reported a significantly higher association between CHB infection and GDM than the other studies analysed here. Since the occurrence of both CHB infection and GDM differs among ethnic groups [18], there may be an ethnic difference in the association between CHB infection and GDM.

GDM is characterised by pancreatic β-cell dysfunction that results in insulin resistance (IR). IR has been shown to be associated with both CHC and CHB infection [22], but CHC related IR was mainly due to steatosis development [37] or fibrosis progression [38], which seems to be insignificant for CHB infection as most pregnant women with CHB infections are asymptomatic or experiencing mild hepatic injury [39].

Routine screening for GDM is recommended near the beginning of the third trimester, and the identification of GDM will depend on the diagnostic criteria and guidelines. For economic, psychological and other reasons, GDM screening is conducted using a risk factor-based selective screening approach in most countries rather than universal screening. These differences in strategies did not seem to affect the non-significant association between CHB infection and the risk of GDM [12-14,16,17], which was further supported by our sub-group and meta-regression analysis. Only one out of the ten homogenous studies reported a positive association between CHB infection and the risk of GDM [7]. This difference can be explained by the fact that the replacement of the Australian standard for GDM diagnosis with the WHO standard would have resulted in the inclusion of individuals with impaired glucose tolerance into the GDM group. Routinely, pregnant women will receive the same screening and are subjected to the same diagnostic criteria in the same study regardless of CHB infection status, so issues of this type seem to play insignificant roles in most of the studies [3,5,8,9,13,14,16,17]. Future studies should use a universal screening strategy together with the new diagnostic criteria established for GDM.

## Conclusions

Our findings imply that GDM is not attributable to CHB infection during pregnancy. Thus, any extra GDM screening for this population is not necessary, which is a commonly accepted practice throughout the world except in Hong Kong, China. This extra screening will likely result in a greater economic burden and additional psychological concerns such as fear or guilt, especially in epidemic areas, like China. Probably, advanced clinical stages of liver diseases of varied aetiologies may be more important in the development of GDM, and the significantly high association between CHB infection and GDM in Iranian pregnant women is worth further study.

## Competing interests

The authors declare that they have no competing interests.

## Authors’ contributions

YZ and YL conceived and coordinated the study and drafted the manuscript. DK and HL participated in the study design, reference search and data analysis. WH and SW participated in the study design and data extraction. DK and AH participated in the quality rating and data analysis. YW and YL participated in the quality rating and the data extraction and analysis. NZ supervised the statistical analysis. All authors read and approved the final manuscript.

## Supplementary Material

Additional file 1: Figure S1 Flow chart describing the paper retrieval and analysis strategies for this meta-analysis. GDM: gestational diabetes mellitus; CHB: chronic hepatitis B infection. Click here for file
