# Supplementary material for: A meta-analysis of the association between gestational diabetes mellitus and chronic hepatitis B infection during pregnancy
Source: BMC Res Notes. 2014 Mar 11;7:139. doi: 10.1186/1756-0500-7-139 (PMC4007522; doi:10.1186/1756-0500-7-139)
Supplement: Additional file 1: Figure S1 — Flow chart describing the paper retrieval and analysis strategies for this meta-analysis. GDM: gestational diabetes mellitus; CHB: chronic hepatitis B infection. [file 1756-0500-7-139-S1.pptx]

## Slide 1
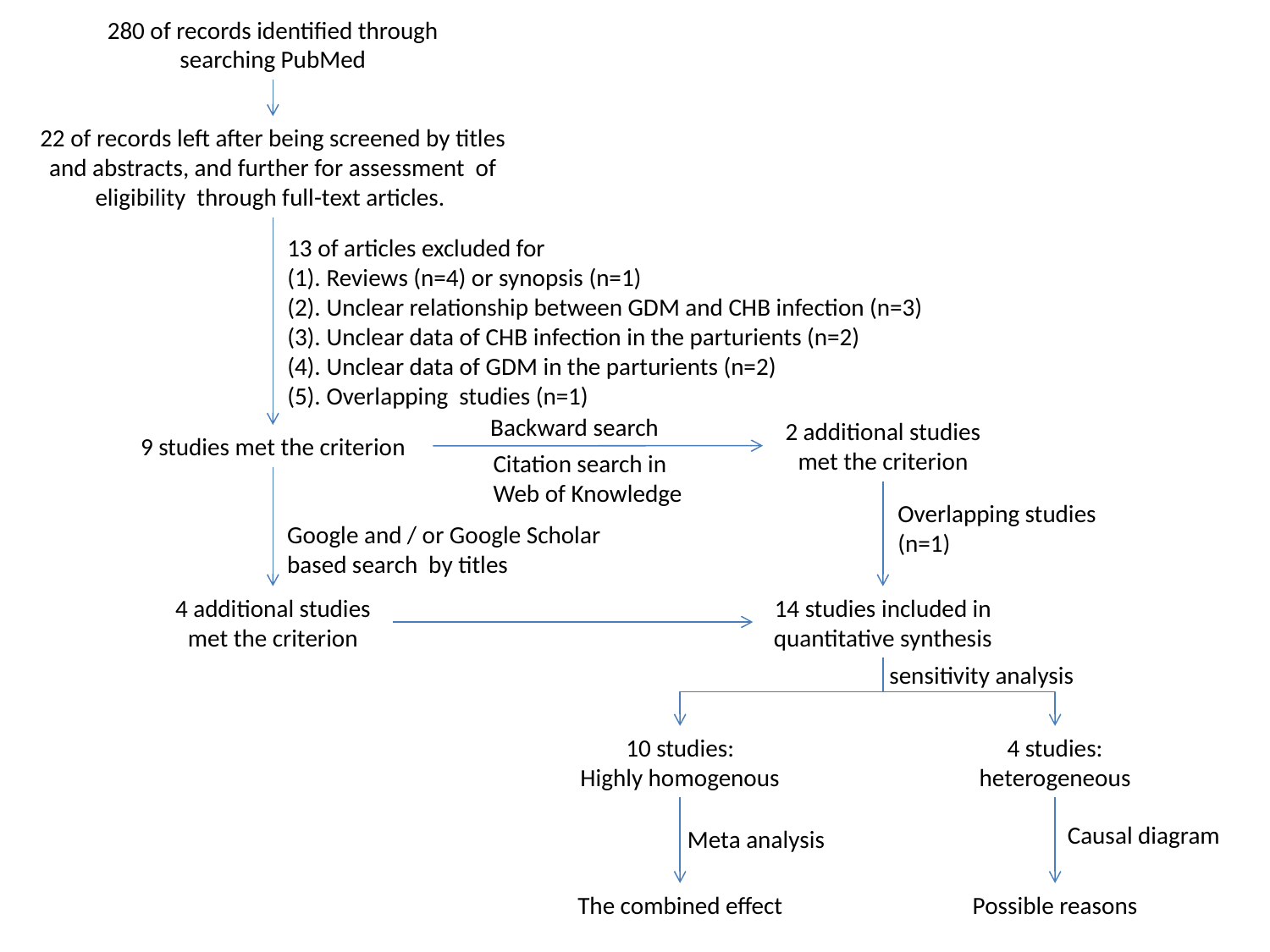

280 of records identified through searching PubMed
22 of records left after being screened by titles and abstracts, and further for assessment of eligibility through full-text articles.
13 of articles excluded for
(1). Reviews (n=4) or synopsis (n=1)
(2). Unclear relationship between GDM and CHB infection (n=3)
(3). Unclear data of CHB infection in the parturients (n=2)
(4). Unclear data of GDM in the parturients (n=2)
(5). Overlapping studies (n=1)
Backward search
2 additional studies met the criterion
9 studies met the criterion
Citation search in
Web of Knowledge
Overlapping studies (n=1)
Google and / or Google Scholar based search by titles
4 additional studies met the criterion
14 studies included in quantitative synthesis
sensitivity analysis
10 studies:
Highly homogenous
4 studies:
heterogeneous
Causal diagram
Meta analysis
The combined effect
Possible reasons
